# Supplementary material for: CENP-E initiates chromosome congression by opposing Aurora kinases to promote end-on attachments
Source: Nat Commun. 2025 Oct 21;16:8537. doi: 10.1038/s41467-025-64148-w (PMC12540835; doi:10.1038/s41467-025-64148-w)
Supplement: Supplementary file 1 — Supplementary Information [file 41467_2025_64148_MOESM1_ESM.pdf]

CENP-E initiates chromosome congression by opposing Aurora kinases to promote end-on attachments

Supplementary Information

Kruno Vukušić*, Iva M. Tolić*
Division of Molecular Biology, Ruđer Bošković Institute, Zagreb, Croatia
*Corresponding authors
E-mail: kvukusic@irb.hr, tolic@irb.hr

Supplementary Table 1. List of reagents, antibodies, cell lines, and software.

Reagent/resource	Reference or source	Identifier or catalog number
**Experimental models**		
hTERT-RPE1 cells stably expressing CENP-A-GFP,	Alexey Khodjakov Lab, Wadsworth Center	N/A
hTERT-RPE1 cells stably expressing both CENP-A-GFP and centrin1-GFP	Alexey Khodjakov Lab, Wadsworth Center	N/A
hTERT-RPE1 cells stably expressing CENP-A-GFP and Mis12-mCherry	Alexey Khodjakov Lab, Wadsworth Center	N/A
hTERT-RPE1 cells stably expressing CENP-A-mCerulean and Mad2-mRuby	Jonathon Pines Lab, Institute for Cancer Research	N/A
U2OS cells induciblely expressing GFP-CENP-E-T422	Marin Barišić Lab, Danish Cancer Institute	N/A
U2OS cells induciblely expressing GFP-CENP-E-WT	Marin Barišić Lab, Danish Cancer Institute	N/A
**Antibodies**		
Rabbit monoclonal Anti-NDC80	Sigma-Aldrich	HPA066330
Mouse monoclonal anti-KIFC1 (M-6)	Santa Cruz	sc-100947
Rabbit polyclonal anti-Kif18a	Bethyl Laboratories	A301-080A
Rabbit anti-Kif4A	Bethyl Laboratories	A301-074A
Rabbit anti-SPINDLY/CCD98	Bethyl Laboratories	A301-354A
Mouse monoclonal anti-KID (B-9)	Santa Cruz	sc-390640
Rat anti-alpha-tubulin YL1/2	Invitrogen	MA1-80017
Human anti-centromere (CREST) protein	Antibodies Incorporated	15-234
Mouse monoclonal anti-Astrin, clone C-1	Merck	MABN2487
Rabbit polyclonal anti-ZW10	Abcam	ab21582

Donkey anti-rabbit IgG Alexa Fluor 647	Abcam	ab150075
Donkey anti-rabbit IgG Alexa Fluor 594	Abcam	ab150064
Donkey anti-mouse IgG Alexa Fluor 594	Abcam	ab150108
Donkey anti-mouse IgG Alexa Fluor 647	Abcam	ab150107
Donkey anti-rat IgG Alexa Fluor 594	Abcam	ab150156
Donkey anti-rat IgG Alexa Fluor 647	Abcam	ab150155
Goat anti-Human DyLight 594	Abcam	ab96909
**Oligonucleotides and other sequence-based reagents**		
Human CENP-E ON-TARGETplus SMART pool siRNA	Dharmacon	L-003252-00-0010
Human PRC1 ON-TARGETplus SMART pool siRNA	Dharmacon	L-019491-00-002
Human custom-made Ndc80/Hec1 siRNA, oligo #1	Dharmacon	sequence: 5’-GAAUUGCAGCAGACUAUUA-3’
Human MISSION esiRNA kntc2 (NDC80), oligo #2	Merck	EHU042171-20UG
Human ON-TARGETplus SMART pool NUF2 siRNA	Dharmacon	L-005289-00-000
Human custom-made 3’UTR HEC1 siRNA, oligo #3	Thermo Fisher Scientific	sequence 5′-CCCUGGGUCGUGUCAGGAA-3′
Human ON-TARGETplus SMART pool KIFC1 (HSET) siRNA	Dharmacon	L-004958-0010
Human KIF4A siRNA	Santa Cruz Biotechnology	sc-60888
Human Kif18A siRNA	Ambion	#Cat 4390825, ID:s37882
Human Kif22/Kid siRNA	Ambion	#Cat 4392420, ID:s7911

Human WAPL ON-TARGETplus SMART pool siRNA	Dharmacon	J-026287-10-0010
Human BUB1B/BuBR1 ON-TARGETplus SMART pool siRNA	Dharmacon	L-004101-00-0005
Human custom-made Spindly siRNA	Sigma-Aldrich	Sequence antisense: 5'-GAAAGGUCUCAAACUGAA-3', dTdT overhangs
Human CENP-E 3'UTR siRNA	Marin Barišić Lab, Danish Cancer Institute	5′-CCACUAGAGUUGAAAGAUA-3′
Control siRNA	Dharmacon	D-001810-10-05
**Chemicals, enzymes and other reagents**		
MG-132	Merck	M7449
Lipofectamine RNAiMAX Reagent	Thermo	13778150
OPTI-MEM medium	Thermo	31985062
DMEM medium	Thermo	11995065
Barasertib	Selleckchem	S1147
GSK-923295	MedChemExpress	HY-10299
Monastrol	MedChemExpress	HY-101071A
ZM-447439	MedChemExpress	HY-10128
MLN8037	MedChemExpress	HY-10971
BAY-320	MedChemExpress	HY-104000
5-Iodotubercidin	MedChemExpress	HY-15424
AZ3146	MedChemExpress	HY-14710
BI 2536	MedChemExpress	HY-50698
Okadaic acid	MedChemExpress	HY-N6785
Ciliobrevin D	MedChemExpress	HY-122632
Fetal bovine serum (FBS)	Thermo-Fisher	10270106
µ-Dish 35 mm, high Glass Bottom	Ibidi	81158
35 mm Dish 1.5 Coverslip 10 mm Glass Diameter Uncoated	MatTek	P35G-1.5-10-C

**Software**		
ImageJ (Fiji)	https://imagej.net/software/fiji/	N/A
Prairie View Imaging Software	Bruker	N/A
Imspector software	Abberior	N/A
ZEN 2.6 software	Zeiss	N/A
MatlabR2021a	MathWorks	
Matlab extension UnivarScatter	https://github.com/manulera/UnivarScatter	N/A
Matlab Statistics Toolbox R14	MathWorks	N/A
Adobe Illustrator CS6	Adobe Systems	N/A
Low Light Tracking Tool (v.0.10) ImageJ plugin	https://imagej.net/plugins/low-light-tracking-tool	N/A
**Microscopy systems**		
Opterra I multipoint scanning confocal microscope system	Bruker	N/A
Expert Line easy3D STED microscope system	Aberior	N/A
LSM 800 confocal laser scanning microscope system	Zeiss	N/A
Lattice Lightsheet 7 microscope system	Zeiss	N/A

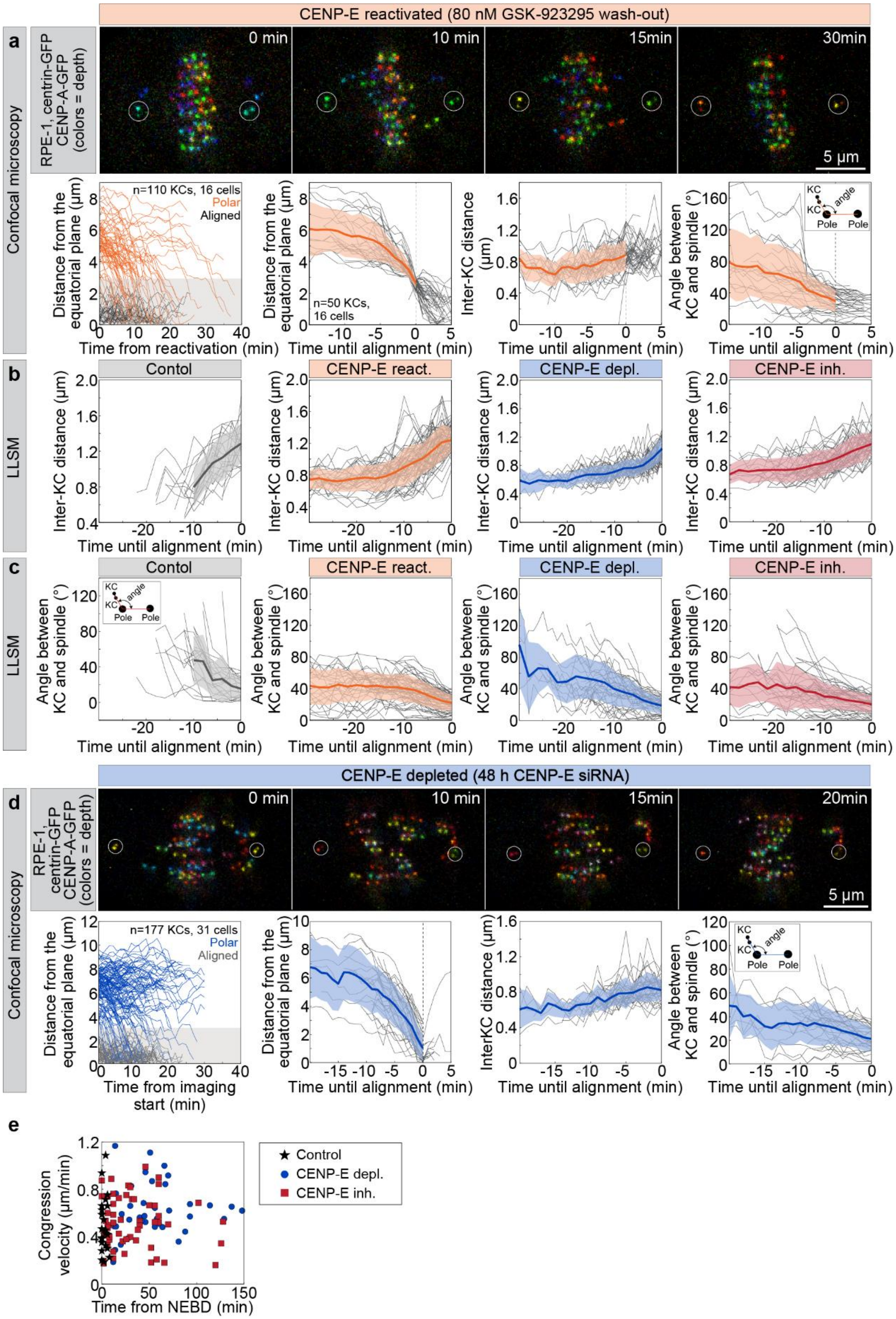

Supplementary Fig. 1. CENP-E activity and mitotic duration do not affect chromosome congression dynamics after initiation in RPE-1 cells. (a) Representative confocal images of an

RPE-1 cell expressing CENP-A-GFP and centrin1-GFP after washout of 80 nM CENP-E inhibitor GSK923295, shown as maximum intensity projections color-coded by depth (color bar in Fig. 1c). Plots show the distance from the equatorial plane (top), interkinetochore distance (inter-KC, middle), and angle between sister kinetochores and the spindle axis, as illustrated in the top-left scheme (bottom), of sister kinetochores for initially polar (orange) pairs, over time from CENP-E inhibitor washout, with means (thick lines) and standard deviations (shaded areas). Centrioles are marked by white circles. In top left graph black lines represent aligned kinetochores at the moment of inhibitor washout. **(b)** Distance between polar kinetochores (inter-KC) until alignment across indicated treatments imaged by lattice light sheet microscopy (LLSM). **(c)** Angle between polar kinetochores and the spindle axis, as depicted in the top-left scheme, until alignment across treatments imaged by LLSM, (**d**) Representative confocal images of an RPE-1 cell expressing CENP-A-GFP and centrin1-GFP after 48h CENP-E depletion, with similar plots for kinetochore distances, inter-KC, and angles, as in (a). (**e**) Congression velocity of polar kinetochore pairs during the last 6 minutes of chromosome congression plotted against time from nuclear envelope breakdown (NEBD) when congression event was initiated, and for treatments indicated. In (a-d) thick lines represent means and shaded areas represent standard deviations. Numbers are given in panels for (a) and (d). For (b) and (c) numbers are given in legends of Fig. 1. All data are pooled from more than three biological replicates. Abbreviations: KC, kinetochore; depl., depleted; inh., inhibited; react., reactivated; NEBD, nuclear envelope breakdown.

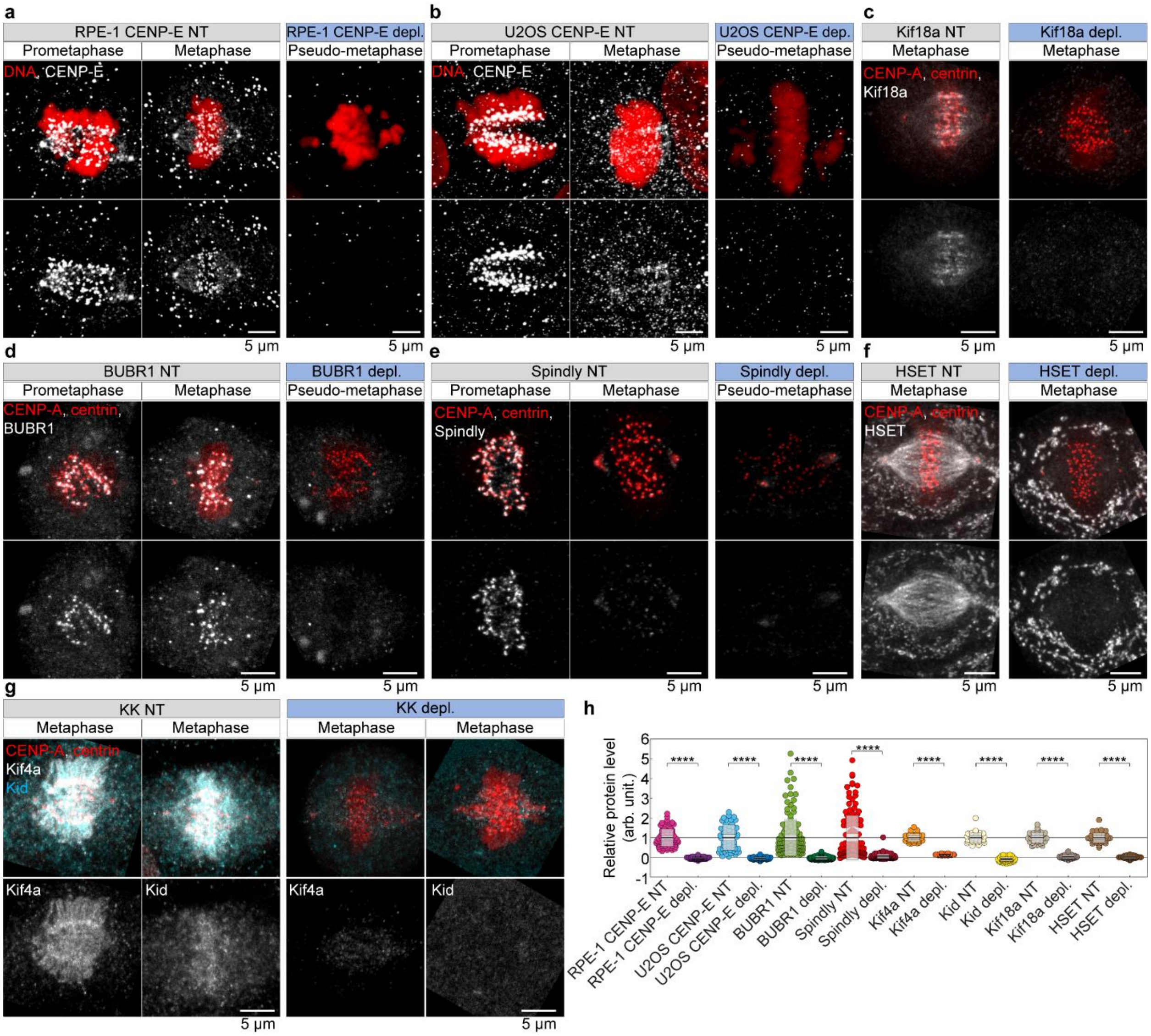

Supplementary Fig. 2. Target-wide assessment of RNA interference efficiency by immunofluorescence. **(a–g)** Representative images of RPE-1 (a, c–g) and U2OS (b) cells treated with non-targeting (NT, grey boxes) or targeting siRNAs (blue boxes), immunostained with antibodies against the indicated proteins. Images show different mitotic stages as labeled. Panels on the left correspond to NT siRNA-treated cells, while panels on the right show cells treated with siRNAs targeting the specific proteins indicated. RPE-1 cells were either wild-type and stained with DAPI (a, red) or expressed CENP-A–GFP and centrin–GFP (c–g, red). U2OS cells were wild-type and stained with DAPI (b, red). Top panels show maximum intensity projections of merged fluorescence channels, and bottom panels show grayscale images of antibody signal for the targeted protein only. **(h)** Quantification of average protein levels normalized to the NT control group. Colored points represent individual cells; black lines show the mean, with light and dark grey areas marking 95% confidence intervals for the mean and standard deviation, respectively. Numbers: 70, 66, 56, 53 cells for CENP-E; 137, 116, 121, 144 kinetochores from 14, 13, 15, and 15 cells for BubR1 and Spindly; and 36, 42, 35, 43, 34, 38, 42, and 36 cells for Kid through HSET, all from at least two independent biological replicates. Statistics: two-tailed ANOVA with post-

hoc Tukey's HSD test. Symbols indicate: ****p < 0.0001; NT, non-targeting; depl., depleted; KK, Kid and Kif4a.

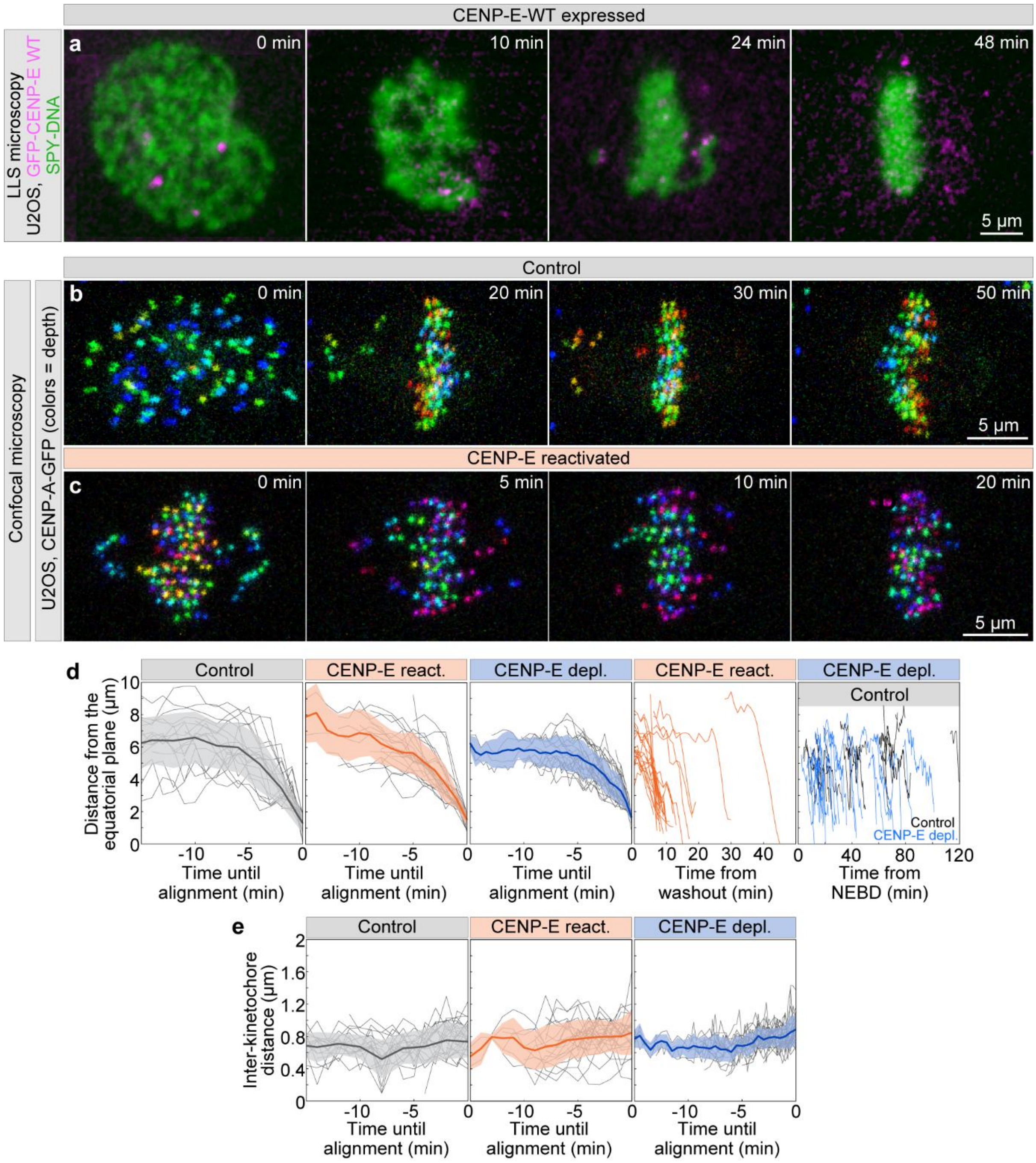

Supplementary Fig. 3. CENP-E activity does not alter chromosome movement dynamics once congression has initiated in U2OS cells. **(a)** Representative image of a U2OS cell expressing GFP–CENP-E wild-type (WT) (magenta) following depletion of endogenous CENP-E, stained with 10 nM SPY650–DNA (green). Time 0 corresponds to the onset of nuclear envelope breakdown (NEBD). **(b, c)** Representative examples of U2OS cells expressing CENP-A–GFP imaged by confocal microscopy at different time points in a control DMSO-treated cell (top) and after washout of 200 nM GSK923295. Time 0 corresponds to the onset of NEBD in (a) or the time of inhibitor washout in (b). Images are shown as maximum intensity projections and color-coded

by depth, as indicated by the color bar in Fig. 1c. **(d, e)** Quantification of the distance from polar kinetochores to the spindle equatorial plate (d) and interkinetochore distance (e) over indicated time points, representing time until alignment, time from inhibitor washout, or time from NEBD, as shown, under different treatment conditions indicated above each graph. Thick lines represent means and shaded areas represent standard deviations. Numbers of cells and independent experiments are given in legend of Fig. 1. Abbreviations: react., reactivated; depl., depleted.

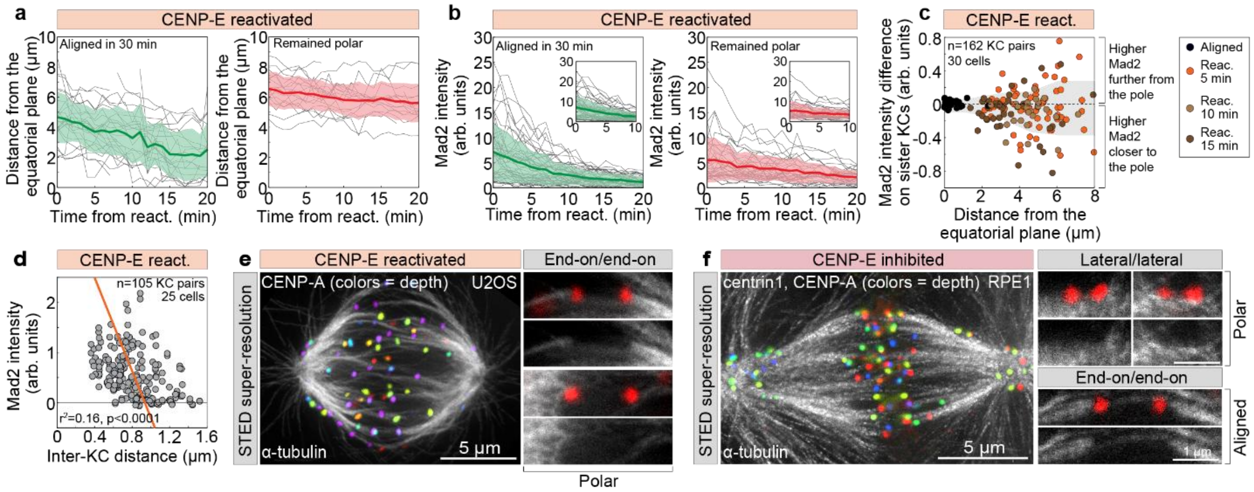

Supplementary Fig. 4. The Mad2 signal on polar kinetochores gradually decreases before the onset of congression movement. **(a, b)** Distance from the equatorial plane (a) and mean Mad2 signal intensity (b) of polar kinetochore pairs over time from washout of the CENP-E inhibitor, shown separately for kinetochore pairs that aligned within 30 minutes (green, left) and pairs that did not align in the same period (red, right). Thick lines represent means and shaded areas represent standard deviations. **(c)** Distance of kinetochore pairs from the equatorial plane plotted against the difference in Mad2 levels between sister kinetochores based on their proximity to the nearest pole under the indicated conditions (right). **(d)** Interkinetochore (inter-KC) distance of sister kinetochore pairs versus mean Mad2 signal intensity with linear regression (line). **(e, f)** Representative spindles from a U2OS cell 8 minutes after CENP-E reactivation (e) and an RPE-1 cell after continuous CENP-E inhibition (f), immunostained for α-tubulin (grey) and imaged by stimulated emission depletion (STED) microscopy. U2OS cells expressed CENP-A-GFP and RPE-1 cells expressed CENP-A-GFP and centrin1-GFP, which are color coded by depth, as specified by the color bar in Fig. 1c (left). Images are maximum intensity projections. Insets show kinetochore pairs with microtubules from different attachment categories as depicted, alongside schematic representations of microtubules (white) and kinetochores (red) for the respective insets (right). Statistics two-tailed t-test. Numbers of cells and independent experiments for (a) and (b) are given in legend of Fig. 2. All data are pooled from at least three independent biological replicates. Abbreviations: react., reactivation; KC, kinetochore; inh., inhibited.

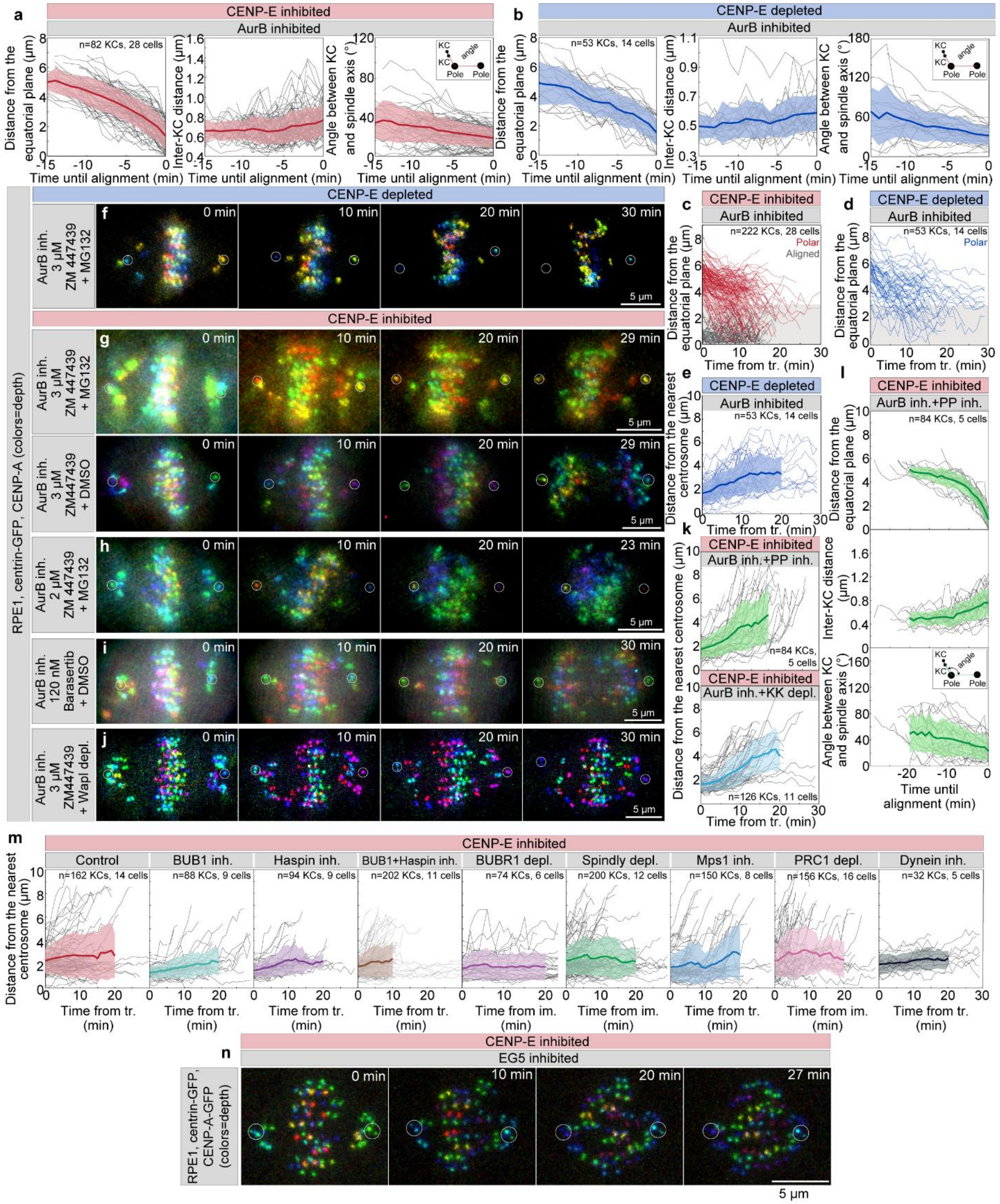

Supplementary Fig. 5. Kinetochore-localized Aurora B constitutes the main barrier to chromosome congression initiation when CENP-E is absent. (a, b) Distance to the equatorial plane (left), interkinetochore distance (middle), and angle between sister kinetochores and spindle axis (right, scheme) over time until alignment after acute Aurora B inhibition (3 µM ZM447439) in CENP-E inhibited (a) or depleted (b) cells. Centrioles circled in white. **(c–e)** Distance to

equatorial plane of initially polar (red/blue) and aligned (black) sister kinetochores over time from Aurora B inhibitor addition in CENP-E inhibited (c) and depleted (d) cells; (e) distance to nearest spindle pole of polar kinetochores in CENP-E depleted cells. **(f–j)** Representative RPE1 cells expressing CENP-A-GFP and centrin1-GFP after treatments: acute Aurora B inhibition with 3 µM ZM447439 in CENP-E depleted (f), in CENP-E inhibited cells ± 20 µM MG132 (g), Aurora B inhibition with 2 µM ZM447439 + MG132 (h), Aurora B inhibition by 120 nM barasertib + DMSO (i), and Aurora B inhibition with ZM447439 + DMSO in WAPL-depleted, CENP-E inhibited cells (j). Centrioles circled. Images are color coded for depth (color bar in Fig. 1c). **(k)** Distance to nearest centrosome of polar kinetochores over time for indicated treatments. **(l)** Distance to equatorial plane (top), inter-KC distance (middle), and angle to spindle axis (bottom) in cells with combined Aurora B (3 µM ZM447439) and protein phosphatase (1 µM Okadaic acid) inhibition under CENP-E inhibition. (**m**) Distance to the nearest centrosome over time for initially polar chromosomes in different treatments. **(n)** Example of RPE1 cell after acute addition of 100 µM Eg5 inhibitor monastrol in CENP-E inhibited cells. Time indicated from treatment. In (a-m) thick lines represent means and shaded areas represent standard deviations. All images are maximum projections color-coded by depth (Fig. 1a color bar). Numbers of cells and kinetochores are given in panels. All data are pooled from at least three independent biological replicates. Abbreviations: KC, kinetochore; depl., depleted; inh., inhibited; react., reactivated; tr., treatment; im., imaging; KK, Kid and Kif4a; PP, protein phosphatases.

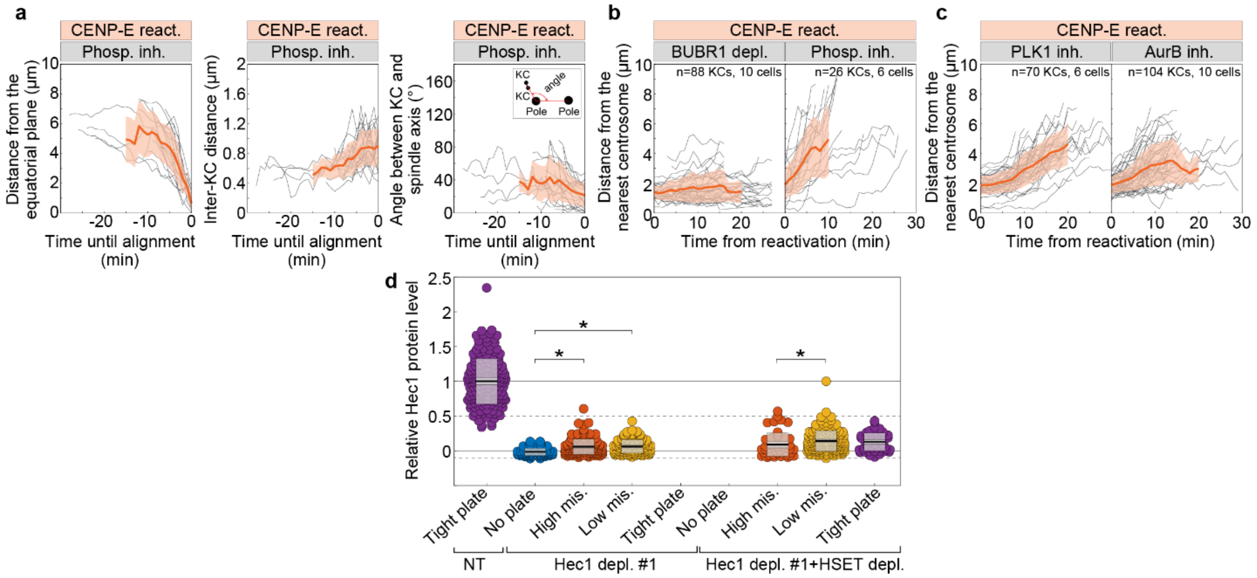

Supplementary Fig. 6. Chromosome congression after CENP-E reactivation occurs independently of protein phosphatase activity. **(a)** Distance to the equatorial plane of initially polar kinetochore pairs (left), interkinetochore distance (middle), and angle between sister kinetochores and the spindle axis (right, scheme) over time until alignment in cells treated with 1 µM Okadaic acid to acutely inhibit protein phosphatases after CENP-E inhibitor washout. **(b, c)** Distance to nearest centrosome of initially polar kinetochore pairs over time from treatments indicated above each graph. In (a-c) thick lines represent means and shaded areas represent standard deviations. **(d)** Average Hec1 levels on kinetochores normalized to non-targeting (NT) controls across treatments and alignment categories as indicated. Colored points represent individual cells; black lines show the mean, with light and dark grey areas marking 95% confidence intervals for the mean and standard deviation, respectively. Numbers for (a-c) are given on panels. Numbers for (d) are given in legend of Fig. 7. All data are pooled from at least three independent biological replicates. Statistics: two-tailed ANOVA with Tukey's HSD post-hoc test. Symbols: * P ≤ 0.05. Abbreviations: KC, kinetochore; inh., inhibited; depl., depleted; react., reactivated; mis., misalignment.
