# Supplementary material for: CENP-E initiates chromosome congression by opposing Aurora kinases to promote end-on attachments
Source: Nat Commun. 2025 Oct 21;16:8537. doi: 10.1038/s41467-025-64148-w (PMC12540835; doi:10.1038/s41467-025-64148-w)
Supplement: Supplementary file 2 — Description of Additional Supplementary Files [file 41467_2025_64148_MOESM2_ESM.pdf]

**Description of Additional Supplementary Files:**

Supplementary Movie 1: Lattice-light sheet microscopy (LLSM)-based assay for largescale live cell imaging.

Supplementary Movie 2: RPE1 cells stably expressing CENP-A-GFP and Centrin1-GFP in control and CENP-E reactivated conditions, imaged by LLSM.

Supplementary Movie 3: RPE1 cells stably expressing CENP-A-GFP and Centrin1-GFP in CENP-E depleted and CENP-E inhibited conditions, imaged by LLSM.

Supplementary Movie 4: RPE1 cells stably expressing CENP-A-mCerulean and Mad2- mRuby following reactivation of CENP-E activity, imaged by confocal microscopy.

Supplementary Movie 5: RPE1 cells stably expressing CENP-A-GFP and Centrin1-GFP in CENP-E inhibited condition following addition of inhibitors of Aurora kinases, and in CENP-E reactivated condition following depletion of BubR1 and addition of inhibitor of phosphatases, imaged by confocal microscopy.
